# Supplementary material for: Interferon-γ Preferentially Promotes Necroptosis of Lung Epithelial Cells by Upregulating MLKL
Source: Cells. 2022 Feb 6;11(3):563. doi: 10.3390/cells11030563 (PMC8833897; doi:10.3390/cells11030563)
Supplement: Supplementary file 1 [file cells-11-00563-s001.zip › cells-1555525-supplementary.pdf]

Figure S1

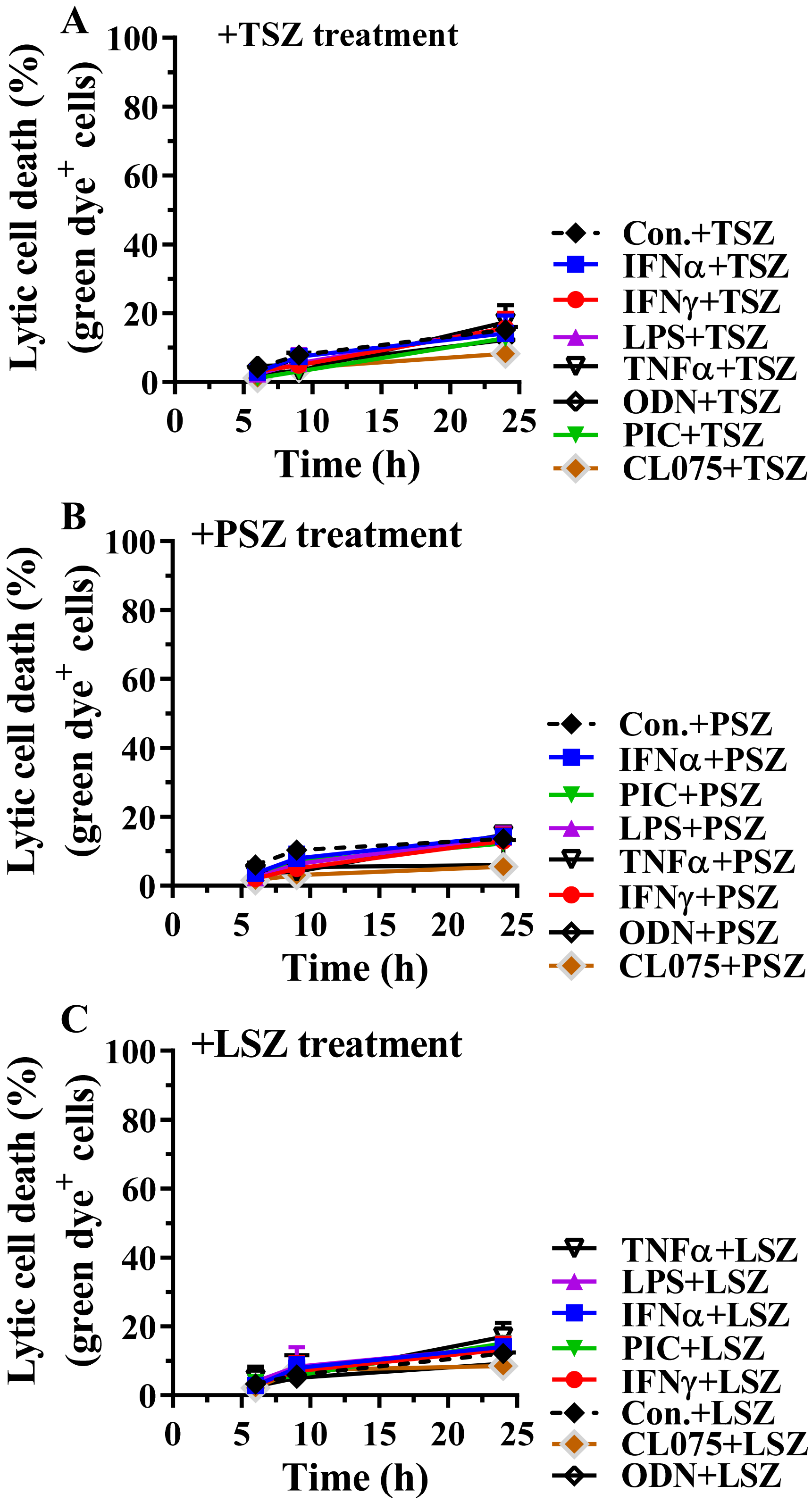

Figure S1. IFNγ and other agonists do not promote necroptosis in lung microvascular endothelial cells. Primary mouse lung vascular endothelial cells were incubated with PBS (Con.), IFNα2 (50 ng/ml), IFNγ (50 ng/ml), TNFα (25 ng/ml), LPS (100 ng/ml), poly(I:C) (PIC, 0.5 μg/ml), CL075 (1 μg/ml) or ODN1585 (1 μM) for 24 h, then treated 0 to 24 h with necroptosis inducers TSZ (**A**), PSZ (**B**), or LSZ (**C**) in the presence of the cyanine dye. The percentage of lytic cell death (green dye^{+} cells) was calculated by comparison to the maximal fluorescence induced by a lysis buffer in each group and are presented as means ± SE (n=3). The orders of each agonist-induced percentage of lytic cell death were listed on the right in each panel.

Figure S2

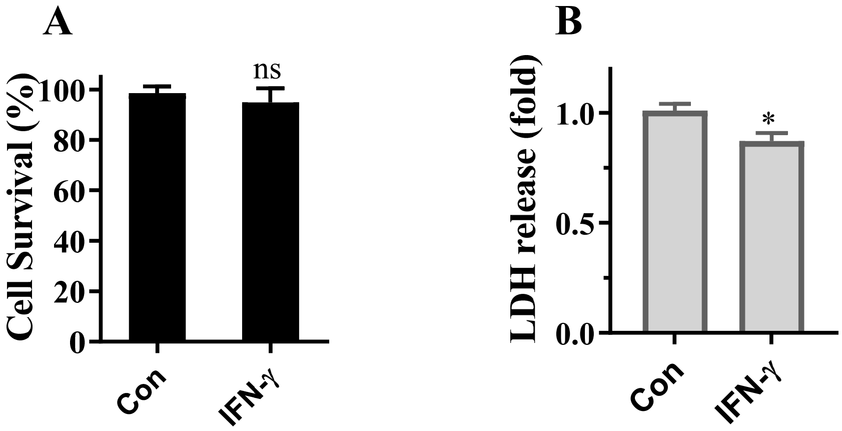

Figure S2. IFNγ alone has no effect on cell survival and a minor effect on LDH release in primary AECs. Primary mouse AECs were incubated with PBS (Con.) or IFNγ (50 ng/ml) for 24 h. **(A)** AEC viability was assessed by MTS assay using CellTiter A_{Queous} reagent and is presented as mean ± SE (n=10). **(B)** LDH activities in cell supernatant were determined and relative changes in LDH are presented as fold ± SE (n=9). Student's t test was performed in (**A, B**). NS, no significance; *p<0.05 versus control (Con.).
